# Fundus-Derived Predicted Age Acceleration in Glaucoma Patients Using Deep Learning and Propensity Score-Matched Controls

**DOI:** 10.3390/jcm14062042

**Published:** 2025-03-17

**Authors:** Masaki Tanito, Makoto Koyama

**Affiliations:** 1 Department of Ophthalmology, Shimane University Faculty of Medicine, Enya 89-1, Izumo 693-8501, Shimane, Japan; 2 Minamikoyasu Eye Clinic, 2-8-30 Minamikoyasu, Kimitsu 299-1162, Chiba, Japan; minamikoyasuganka@gmail.com

**Keywords:** glaucoma, deep learning, fundus photographs, predicted age acceleration, retinal age gap, oculomics, biological clock

## Abstract

**Background/Objectives:** Glaucoma, a leading cause of irreversible blindness, has been associated with systemic and ocular aging processes. This study aimed to investigate the relationship between glaucoma and accelerated biological aging using fundus-derived age prediction. Additionally, the role of systemic factors and retinal vascular changes in this association was explored. **Methods:** A total of 6023 participants, including 547 glaucoma patients and 547 matched controls, were analyzed. Fundus-derived predicted age was assessed using a deep learning model (EfficientNet). Systemic factors such as BMI, blood pressure, lipid profiles, liver function markers, glucose levels, and retinal vascular changes (Scheie classifications) were analyzed. Statistical comparisons and multivariate regression analyses were performed to evaluate the impact of glaucoma on predicted age acceleration, adjusting for age, gender, and systemic factors. **Results:** Glaucoma was significantly associated with higher predicted age acceleration (prediction difference: −1.5 ± 4.5 vs. −2.1 ± 4.5 years; *p* = 0.040). Multivariate regression confirmed that glaucoma independently influenced predicted age (*p* = 0.021) and prediction difference (*p* = 0.021). Among systemic factors, γ-GTP was positively associated with prediction difference (*p* = 0.036), while other factors, such as BMI, blood pressure, and glucose levels, showed no significant association. Retinal vascular changes, including hypertensive and sclerotic changes (Scheie classifications), were significantly more prevalent in glaucoma patients and correlated with predicted age acceleration. **Conclusions:** Glaucoma is associated with accelerated biological aging, as indicated by fundus-derived predicted age. Systemic factors such as γ-GTP and retinal vascular changes may play contributory roles. Fundus-derived predicted age holds promise as a non-invasive biomarker for monitoring systemic aging. Further longitudinal studies are warranted to establish causal relationships and enhance clinical applications.

## 1. Introduction

Glaucoma is a leading cause of irreversible blindness worldwide, characterized by progressive optic neuropathy and visual field loss. While intraocular pressure (IOP) remains a primary modifiable risk factor, myopia and older age have also been identified as significant contributors to the development of glaucoma [1]. The Tajimi Study, a population-based survey in Japan, highlighted the strong association of these factors, emphasizing the need for comprehensive risk assessments in glaucoma management [2]. Similarly, the Barbados Eye Studies and other population-based research have demonstrated that advancing age is a critical risk factor for both the onset and progression of glaucoma [3]. Recent studies have also demonstrated that deep learning models trained on fundus photographs can accurately diagnose glaucoma, providing high sensitivity and specificity [4,5]. These findings underscore the potential of deep learning in advancing glaucoma screening and diagnosis.

Advances in imaging technology and machine learning have also facilitated the emergence of oculomics, a field that explores the use of ocular biomarkers to assess systemic health and disease risk [6,7,8,9]. Oculomics has demonstrated potential in linking ocular characteristics, such as fundus vascular changes, with systemic conditions, including cardiovascular diseases, neurodegenerative disorders, and diabetes [6,7,8,9,10]. The integration of deep learning into oculomics has further enhanced its utility, enabling precise analyses of fundus photographs for both ocular and systemic health assessments [11,12].

Among recent advancements, fundus-derived age prediction—a novel biomarker extracted using deep learning—has emerged as a promising tool for assessing both systemic and ocular health. Discrepancies between predicted age and chronological age, known as predicted age acceleration or retinal age gap, may indicate underlying pathological processes, including those related to aging and systemic diseases such as future stroke risk and mortality risk [13,14] as well as ocular conditions like diabetic retinopathy and glaucoma [15,16]. Consequently, age estimation based on fundus photographs holds significant potential as a biological clock. However, it remains unclear whether glaucoma is specifically associated with an acceleration in biological aging, as reflected in fundus-derived predicted age.

This study aims to determine whether glaucoma is associated with accelerated biological aging by comparing fundus-derived predicted age between glaucoma patients and matched controls. Additionally, the analysis includes an investigation of potential factors influencing age acceleration, such as fundus vascular changes and systemic parameters, to better understand the mechanisms underlying this association.

## 2. Materials and Methods

### 2.1. Subjects

This study adhered to the principles of the Declaration of Helsinki and the Ethical Guidelines for Life Science and Medical Research Involving Human Subjects established by the Government of Japan. The study protocol was reviewed and approved by the Medical Research Ethics Committee of Shimane University Faculty of Medicine (IRB No. KS20230719-3; latest approval date: 22 April 2024). Instead of obtaining signed consent from participants, information about this study was published on the participating institution’s website, and an opt-out opportunity was provided.

**Figure 1:**
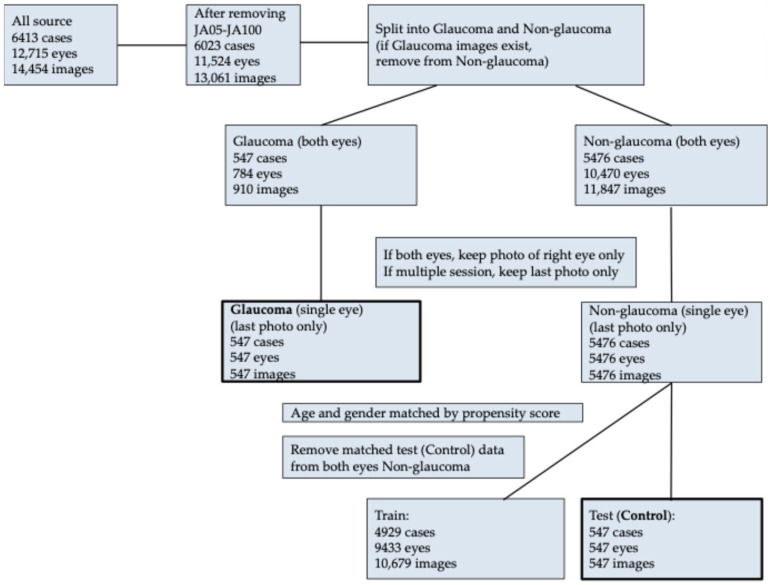
Flowchart of the data selection process.

This study included 6413 participants (12,715 eyes, 14,454 images) who underwent health screening conducted by Japan Agricultural Shimane Kosei-ren (Izumo, Japan) between April 2023 and March 2024. At least one fundus photograph in color was captured for each participant using a non-mydriatic fundus camera (CR-DG 10, Canon, Tokyo, Japan) with a capture angle of 45 degrees. The images, with a resolution of 2400 × 1600 pixels, were saved in JPEG format. The data selection process is illustrated in Figure 1.

All fundus photographs were evaluated during the health screening by a single examiner (M.T.), who assigned diagnostic codes (JA01–JA100). The diagnosis of glaucoma was based on the Japan Glaucoma Society Guidelines for Glaucoma [17]. Assessment included a comprehensive evaluation of glaucomatous features, such as focal or generalized rim thinning, an enlarged cup-to-disc ratio with or without laminar dot sign, retinal nerve fiber layer defects with margins at the optic disc, disc hemorrhages, and peripapillary atrophy. Cases involving optic nerve anomalies like hypoplasia or optic pits were excluded. Images associated with fundus diseases potentially causing visual impairment were excluded based on diagnostic codes, resulting in the selection of 6023 participants, 11,524 eyes, and 13,061 images. Exclusion codes included JA05 (diabetic retinopathy), JA08 (macular degeneration), JA09 (retinal vein occlusion), JA10 (retinal hemorrhage), JA11 (soft exudates), JA13 (other), JA17 (optic nerve hypoplasia), JA14 (suspected cataract), JA18 (pathologic myopia fundus), JA19 (retinal arteriole microaneurysms), JA20 (retinal photocoagulation scars), JA21 (retinochoroidal atrophy), JA99 (uninterpretable), and JA100 (unexamined). Blurred images were excluded using codes JA14 and JA99.

From the selected images, photographs were categorized into Glaucoma (JA01) and Non-Glaucoma groups. For each participant, one eye image was selected. If both eyes were available, the right eye was chosen; if multiple images of the same eye were captured, the most recent image was used. For participants with unilateral glaucoma, the contralateral eye was excluded from the Non-Glaucoma group. This process resulted in 547 images from 547 eyes of 547 participants in the Glaucoma group and 5476 images from 5476 eyes of 5476 participants in the Non-Glaucoma group. Using propensity score matching, 547 participants from the Non-Glaucoma group were matched 1:1 with the Glaucoma group to create a control group. Propensity scores were calculated using a nominal logistic regression model with age and gender as independent variables. Nearest neighbor matching with a caliper coefficient (alpha = 0.2) was applied. Unmatched Non-Glaucoma participants and the contralateral eyes of matched Non-Glaucoma participants were excluded. The final dataset for training the age prediction algorithm consisted of 4929 participants, 9433 eyes, and 10,679 images from the unmatched Non-Glaucoma group.

### 2.2. Development of an Age Prediction Model from Fundus Photographs

The average age of the training dataset was 64.6 ± 12.8 years, with 47% male and 53% female. In this study, we employed the EfficientNet model “tf_efficientnet_b5.ns_jft_in1k” as the backbone for the age prediction task. EfficientNet, a convolutional neural network (CNN), is widely recognized for its performance and efficiency [18]. The pre-trained weights were obtained from the version hosted at https://huggingface.co/timm/tf_efficientnet_b5.ns_jft_in1k (accessed on 4 January 2025), which was trained on the ImageNet-1k and unlabeled JFT-300M datasets using the Noisy Student semi-supervised learning approach. We resized all input images to 456 × 456 pixels and applied several data augmentation techniques, including random rotation, random horizontal flipping, random vertical flipping, and random cropping, to enhance the model’s robustness and generalizability. The model was fine-tuned on the training dataset using a batch size of 8. The learning rate was initially set to 2 × 10^−4^ and gradually increased to 1 × 10^−3^ over the first three epochs. It was then decreased back to 2 × 10^−4^ over the following 19 epochs, for a total of 22 training epochs. The model’s output was directly connected to the age prediction without any intermediate hidden layers. We optimized the model by minimizing the Mean Squared Error between the predicted age and the true age. We used the AdaBelief optimizer, and all deep learning experiments were implemented in Python (version 3.11.2) with PyTorch (version 2.01).

### 2.3. Statistical Analysis

The following variables were collected from the health screening data: date of birth, date of fundus photography, gender, body mass index (BMI), systolic blood pressure (SBP), diastolic blood pressure (DBP), and results of peripheral blood tests, including high-density lipoprotein cholesterol (HDL-C), low-density lipoprotein cholesterol (LDL-C), glutamic oxaloacetic transaminase (GOT), glutamic pyruvic transaminase (GPT), gamma-glutamyl transpeptidase (γ-GTP), glucose, and glycated hemoglobin (HbA1c). Additionally, the Scheie grading score was used to evaluate retinal vascular changes [19,20]. This method assesses hypertensive changes (H0–4) and sclerotic changes (S0–4) based on the appearance of blood vessels in fundus photographs. Other blood test results, urine test parameters, and medical history questionnaire data were not collected due to missing values. The propensity score was calculated using age truncated to integer values. True age was determined by calculating the difference between the date of birth and the date of fundus photography. Prediction difference was defined as the predicted age minus the true age, with both true age and prediction difference treated as continuous variables without truncation. Comparisons between the Glaucoma and control groups were conducted using the *t*-test or Fisher’s exact probability test. To adjust for the effects of age, gender, and other systemic factors, multiple regression analysis was performed. The variance inflation factor (VIF) was applied to detect multicollinearity during the regression analysis. Propensity score calculations and statistical analyses targeting the Glaucoma and control groups were performed using JMP Pro version 17.2 (SAS Institute, Inc., Cary, NC, USA). A *p* value of <0.05 was considered statistically significant. For English proofreading, ChatGPT-4 (OpenAI, San Francisco, CA, USA) was employed to refine the manuscript.

## 3. Results

The comparison of baseline characteristics between the Glaucoma and control groups is summarized in Table 1. Both groups consisted of 547 eyes from 547 subjects, with no significant differences in age (70.2 ± 9.5 vs. 70.2 ± 9.4 years; *p* = 0.99) or gender distribution (male: 51%, female: 49% in both groups; *p* > 0.99). BMI was comparable between the Glaucoma and control groups (*p* = 0.31). Similarly, there were no significant differences in SBP (*p* = 0.67), DBP (*p* = 0.09), or lipid profiles, including HDL-C, LDL-C, and TG (*p* = 0.87, 0.77, and 0.16, respectively). Liver function markers, such as GOT, GPT, and γ-GTP, also showed no significant differences (*p* = 0.44, 0.24, and 0.48, respectively). Regarding glucose metabolism, fasting glucose and HbA1c levels were not significantly different between the groups (*p* = 0.80 and 0.47, respectively). Overall, univariate analysis revealed no significant differences in any of the evaluated parameters between the Glaucoma and control groups.

The comparison of true age, predicted age, and prediction difference between the Glaucoma and control groups is summarized in Table 2. True age was similar between the Glaucoma and control groups. Predicted age also showed no significant difference (68.7 ± 8.8 vs. 68.1 ± 8.1 years; *p* = 0.27). However, the predicted age in the Glaucoma group tended to be slightly older compared to the control group. In both groups, the predicted age was younger than the true age, resulting in a negative prediction difference. The prediction difference was significantly larger in the control group compared to the Glaucoma group (−2.1 ± 4.5 vs. −1.5 ± 4.5 years; *p* = 0.040), indicating that glaucoma was associated with a higher predicted age relative to the true age.

Multivariate regression analysis, adjusting for age and gender, revealed that glaucoma had a significant effect on both predicted age and prediction difference (*p* = 0.021 for both, Table 3). For predicted age, the estimate for the Glaucoma group relative to the control group was 0.28 (*p* = 0.021), indicating that glaucoma was associated with a higher predicted age compared to the control group, even after adjusting for age and gender. Age had a strong positive effect on predicted age (estimate: 0.78/year, *p* < 0.0001), whereas gender showed no significant effect (*p* = 0.18). For prediction difference, the Glaucoma group had a significantly higher estimate compared to the control group (estimate: 0.28, *p* = 0.021). Age had a negative effect on prediction difference (estimate: −0.22/year, *p* < 0.0001), while gender remained nonsignificant (*p* = 0.18). These results suggest that glaucoma significantly influences predicted age and prediction difference, even after adjusting for age and gender.

**Figure 2:**
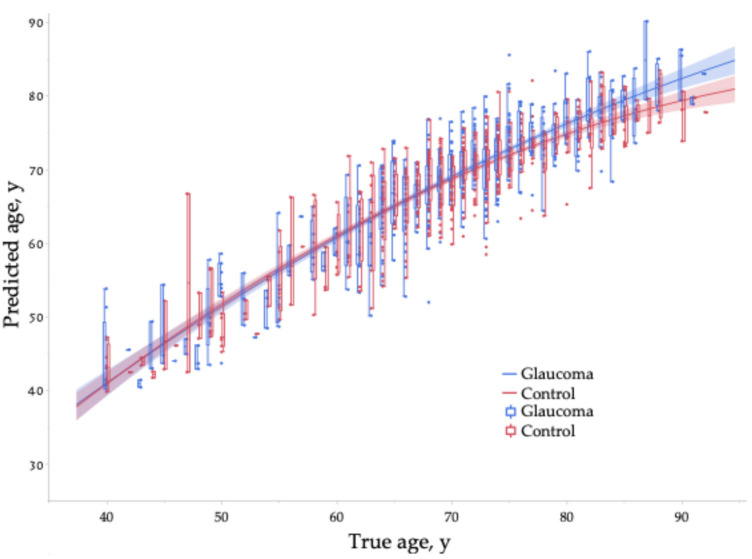
Relationship between true age and predicted age. The line represents a second-order regression curve, and the shaded area indicates the 95% confidence interval of the regression line. Box-and-whisker plots are calculated for each age group.

Figure 2 illustrates the relationship between true age and predicted age. The line represents a second-order regression curve, and the shaded area indicates the 95% confidence interval of the regression line. The regression curves for the Glaucoma and control groups overlap in younger age ranges but begin to diverge from around 65 to 70 years and older, with the Glaucoma group being predicted as older.

To further investigate the impact of glaucoma on predicted age and prediction difference, additional subgroup analyses were conducted based on age quartiles. The comparison of predicted age and prediction difference between the Glaucoma and control groups across age quartiles is summarized in Table 4. In the youngest age quartile (Q1, ≤66 years), there were no significant differences in predicted age (*p* = 0.78) or prediction difference (*p* = 0.62) between the Glaucoma and control groups. Similarly, in the second quartile (Q2, 66–71 years), both predicted age (*p* = 0.68) and prediction difference (*p* = 0.67) showed no significant group differences. However, in the third quartile (Q3, 71–75 years), the predicted age was significantly older in the Glaucoma group compared to the control group (71.9 ± 3.9 vs. 70.8 ± 3.3 years; *p* = 0.015). The prediction difference also showed a significant group difference (−1.6 ± 3.8 vs. −2.6 ± 3.2 years; *p* = 0.013). In the oldest quartile (Q4, >75 years), the Glaucoma group was again predicted to be significantly older than the control group (76.6 ± 4.4 vs. 75.2 ± 3.7 years; *p* = 0.015), with a more pronounced difference in prediction difference (−4.5 ± 4.0 vs. −5.8 ± 3.8 years; *p* = 0.0055). These results indicate that the effect of glaucoma on predicted age and prediction difference becomes more pronounced in older age groups, suggesting an age-dependent influence of glaucoma on the prediction model.

To evaluate the impact of systemic parameters on the age prediction difference, a multivariate regression analysis was performed. The results are summarized in Table 5. Glaucoma was significantly associated with a higher prediction difference compared to the control group (estimate: 0.29/year, *p* = 0.019). Age was the most influential parameter, with a significant negative association with prediction difference (estimate: −0.22/year, *p* < 0.0001). Among other parameters, γ-GTP showed a weak but significant positive association with the prediction difference (estimate: 0.01, 95% CI: 0.00, 0.01; *p* = 0.036; β = 0.07). However, other variables, including BMI, blood pressure (SBP and DBP), lipid profiles (HDL-C, LDL-C, and TG), liver enzymes (GOT and GPT), glucose, and HbA1c, did not show significant associations (*p* > 0.05). Variance inflation factors (VIFs) for all variables were below 3.5, indicating no substantial multicollinearity. These results suggest that glaucoma and age are the primary factors influencing the age prediction difference, with additional minor contributions from γ-GTP.

Finally, to analyze the impact of local ocular factors, fundus vascular changes between the Glaucoma and control groups are summarized in Table 6. Regarding hypertensive changes (Scheie H classification), the distribution significantly differed between the two groups (*p* < 0.0001). In the Glaucoma group, the majority of subjects were classified as grade 1 (65.1%), while 29.1% were classified as grade 0, and 5.9% as grade 2. In contrast, the control group had a higher proportion of subjects in grade 0 (43.7%) and fewer in grade 2 (2.6%). For sclerotic changes (Scheie S classification), the distribution also showed a significant difference between groups (*p* = 0.03). Most subjects in both groups were classified as grade 1 (84.5% in the Glaucoma group and 79.7% in the control group). However, the Glaucoma group had a slightly higher proportion of subjects in grades 2 (3.5%) and 1 (84.5%), compared to the control group (2.6% and 79.7%, respectively). These results suggest that glaucoma is associated with a higher prevalence of hypertensive and sclerotic vascular changes in the fundus, as evidenced by the Scheie H and S classifications.

**Figure 3:**
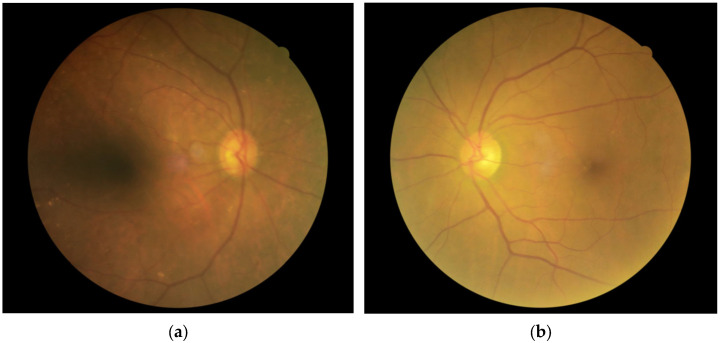
Representative fundus photographs (matching pair No. 27). (**a**) Glaucoma. Female, True age = 73.000001907349, Predicted age = 77.803656458855, Prediction difference = +4.8036545515. (**b**) Control. Female, True age = 73.000001907349, Predicted age = 72.194281220436, Prediction difference = −0.805720687.

Representative fundus photographs are shown in Figure 3a (glaucoma) and Figure 3b (control) for matching pair No. 27. In this matching pair, the prediction difference was +4.8036545515 years for the glaucoma eye, whereas it was −0.805720687 years for the control eye. In the glaucoma eye (Figure 3a), notable findings included marked narrowing and sclerotic changes of the retinal arteries near the inferior-temporal nerve fiber layer defects. Drusen were observed in both the glaucoma and control eyes, but they were more prominent in the glaucoma eye.

## 4. Discussion

This study demonstrated that glaucoma is associated with accelerated biological aging, as reflected by fundus-derived predicted age. The predicted age acceleration, defined as the difference between the predicted age and the chronological age, was significantly greater in the Glaucoma group compared to matched controls. These findings suggest that glaucoma may be linked to systemic and ocular aging processes, which could contribute to its pathophysiology and progression [21,22].

Interestingly, the prediction difference was negative in both the Glaucoma and control groups, indicating that the predicted age was consistently younger than the true chronological age. This phenomenon can likely be attributed to the characteristics of the prediction model used in this study. The training group for the model had a mean age of 64.6 years, while the test group, consisting of both glaucoma and control participants, had a higher mean age of 70.2 years. This age discrepancy suggests that the deep learning model was trained in a way that led to underestimation of age for older individuals. Such a bias may occur when the training dataset underrepresents older age ranges, resulting in a model that systematically predicts younger ages for older test subjects. However, several measures were taken to ensure that the observed group differences in prediction difference are attributable to glaucoma rather than age-related bias. First, the test groups were carefully age-matched between glaucoma and control participants using propensity score matching. Second, the impact of age was rigorously adjusted through multivariate analysis, which confirmed that glaucoma independently contributes to predicted age acceleration after accounting for the influence of age. These methodological precautions strengthen the conclusion that the observed differences reflect intrinsic biological aging processes associated with glaucoma.

Additionally, our age prediction model benefited from a systematic hyperparameter tuning process. Specifically, we began by splitting the original training dataset into a 9:1 ratio (training/validation) to optimize learning rates, batch sizes, data augmentation strategies, and the optimizer. We also conducted preliminary comparisons of multiple architectures—including EfficientNet, SWIN Transformer, and Eva-02—before selecting EfficientNet for its favorable balance of predictive accuracy and computational requirements in this context. After determining the most suitable hyperparameters, we retrained the final model on the entire training dataset (i.e., without maintaining a separate validation split). Although we do not present each intermediate validation result here, these steps may have contributed to the model’s performance in predicting fundus-derived age in our study.

Various hallmarks of aging have been identified through accumulating basic and clinical research [23]; however, a unifying theory or key molecule that comprehensively explains aging has yet to be discovered. It has been reported that systemic factors can influence glaucoma progression, including age-related visual field deterioration and IOP elevation [24,25]. In this study, the pronounced detection of predicted age acceleration in the Q3 and Q4 age groups further highlights the close relationship between glaucoma and aging. Conversely, the lack of significant group differences in younger participants suggests that detecting age acceleration through fundus photographs does not yet provide sufficient evidence for its association with future glaucoma development.

In this dataset, variables such as BMI, blood pressure, lipid profile, and diabetes markers showed no impact on age prediction. Interestingly, γ-GTP was the only factor significantly associated with the prediction difference. A longitudinal analysis of health check-up data in Japan previously reported that elevated γ-GTP levels were related to age-associated increases in IOP [26]. However, since IOP data were missing in our dataset, the impact of γ-GTP on IOP remains unclear. The potential role of γ-GTP in glaucoma pathophysiology is not yet well understood and warrants further investigation. In this study, hypertensive and sclerotic changes in retinal vessels were more pronounced in glaucoma eyes compared to control eyes. Previous analyses of stereo fundus photographs in glaucoma eyes reported that the mean central retinal arteriolar equivalent and central retinal venular equivalent—parameters used to evaluate retinal vascular diameter—narrowed with aging [27]. Moreover, these parameters were also found to be associated with glaucomatous optic nerve head changes [27]. Therefore, the predicted age acceleration observed in fundus photographs may reflect underlying retinal vascular changes. The deposition of drusen in the fundus is a well-documented age-related phenomenon [28], suggesting that drusen accumulation could potentially influence age prediction. Although this study did not quantify drusen, future analyses focusing on the relationship between drusen and glaucoma markers may provide valuable insights into glaucoma assessment. Based on these findings, it can be inferred that our age prediction model assesses not only changes in the optic nerve head but also the aging of the vasculature and the retina itself. However, the specific image parameters that our model actually recognizes remain a black box. This will be an important subject for future research.

Biological age acceleration assessed through epigenomic markers has been reported to correlate with various diseases [29]. Fundus-derived biological age prediction offers a distinct advantage as a non-invasive approach that can be performed using widely available imaging equipment, unlike epigenomic analyses. Fundus-derived predicted age acceleration has the potential to serve as a novel biomarker for identifying individuals at higher risk of glaucoma progression. By integrating deep learning with fundus imaging, clinicians could monitor not only the structural changes characteristic of glaucoma but also systemic health factors that may contribute to disease progression. This approach aligns with the growing emphasis on precision medicine and the application of oculomics in clinical practice.

While this study provides valuable insights, several limitations should be noted. The cross-sectional design does not allow for causal inferences regarding the relationship between glaucoma and predicted age acceleration. Longitudinal studies are required to clarify whether predicted age acceleration precedes or results from glaucoma progression. This study lacked data on key indicators associated with glaucoma diagnosis and severity, such as visual acuity, IOP, visual field, and gonioscopic findings. Additionally, as the dataset included many early-stage glaucoma cases, the reported predicted age acceleration might have been underestimated. Data on glaucoma treatment history and systemic comorbidities were also incomplete, limiting their inclusion in the analysis. Another limitation is that the evaluation of fundus photographs was performed by a single examiner. However, this examiner has extensive experience in fundus photograph assessment, suggesting minimal variability in their judgments. Furthermore, the study population was derived from a single geographic region, which may restrict the generalizability of the findings to other populations. While the deep learning models used in this study provide robust predictions, their interpretability remains limited, necessitating further investigation into the underlying biological mechanisms.

Future studies should aim to validate these findings across diverse populations and investigate the longitudinal relationship between predicted age acceleration and glaucoma progression. The progression speed of glaucoma varies significantly depending on its subtype. Therefore, a case-control study focusing on hospital-based glaucoma cases with clearly defined subtypes and severity would be valuable. Incorporating additional ocular biomarkers, such as retinal nerve fiber layer thickness and vascular parameters, into the analysis could further elucidate the systemic and ocular factors involved in glaucoma-related aging. This study highlights the potential of fundus-derived predicted age as a biomarker for glaucoma-associated aging and emphasizes the significance of systemic and vascular factors in the disease’s pathophysiology. By advancing the integration of deep learning and oculomics, this research lays the groundwork for incorporating biological aging markers into glaucoma management, offering a promising direction for future personalized medicine approaches. The findings of this study do not immediately translate into direct clinical applications. However, advancing our understanding of aging, a major factor in the onset and progression of glaucoma, may contribute to future glaucoma treatments.

## 5. Conclusions

This study revealed that glaucoma is associated with accelerated biological aging, as indicated by fundus-derived age prediction. Despite systematic underestimation of age by the deep learning model, the significant difference in predicted age acceleration between glaucoma patients and matched controls underscores its potential as a biomarker for glaucoma-related systemic aging. Integrating fundus imaging with deep learning offers promising avenues for advancing glaucoma diagnosis and management, highlighting the importance of further research into the mechanisms linking glaucoma and biological aging.

## Figures and Tables

**Table 1:** Comparison of background parameters between the Glaucoma and control groups using univariate analysis.

Parameter	Glaucoma		Control		*p* Value
	Mean ± SD or N (%)	95% CI or N (%)	Mean ± SD or N (%)	95% CI or N (%)	
N	547 eyes	547 subjects	547 eyes	547 subjects	-
Age, y	70.2 ± 9.5	69.4, 71.0	70.2 ± 9.4	69.4, 71.0	0.99
Gender, N (%)	Male, 279 (51)	Female, 268 (49)	Male, 280 (51)	Female, 267 (49)	>0.99
BMI, kg/m^2^	22.6 ± 3.4	22.3, 22.9	22.8 ± 3.6	22.5, 23.1	0.31
SBP, mmHg	131.0 ± 17.4	129.5, 132.4	132.9 ± 18.0	131.4, 134.4	0.67
DBP, mmHg	78.1 ± 11.0	77.2, 79.1	79.2 ± 10.7	78.3, 80.1	0.09
HDL-C, mg/dL	66.3 ± 17.4	64.9, 67.8	66.5 ± 17.8	65.0, 68.0	0.87
LDL-C, mg/dL	117.8 ± 30.8	115.2, 120.4	118.3 ± 28.1	115.9, 120.7	0.77
TG, mg/dL	113.1 ± 109.3	103.9, 122.3	105.6 ± 58.1	100.8, 110.5	0.16
GOT, U/L	24.2 ± 8.5	23.5, 24.9	24.6 ± 10.6	23.7, 25.5	0.44
GPT, U/L	20.3 ± 9.9	19.4, 21.1	21.2 ± 14.6	19.9, 22.4	0.24
γ-GTP, U/L	37.5 ± 48.4	22.5, 41.6	40.0 ± 66.4	34.4, 45.6	0.48
Glucose, mg/dL	100.2 ± 18.4	98.7, 101.7	99.9 ± 19.5	98.3, 101.5	0.80
HbA1c, %	6.0 ± 0.6	5.9, 6.0	5.9 ± 0.6	5.9, 6.0	0.47

*p* values are calculated by *t*-test for continuous variables and by Fisher’s exact probability test for the categorical variable. SD, standard deviation; CI, confidence interval; BMI, body mass index; SBP, systolic blood pressure; DBP, diastolic blood pressure; HDL-C, high-density lipoprotein cholesterol; LDL-C, low-density lipoprotein cholesterol; GOT, glutamic oxaloacetic transaminase; GPT, glutamic pyruvic transaminase; γ-GTP, gamma-glutamyl transpeptidase; HbA1c, glycated hemoglobin.

**Table 2:** Comparison of true age, predicted age, and prediction difference between the Glaucoma and control groups using univariate analysis.

Parameter	Glaucoma		Control		*p* Value
	Mean ± SD	95% CI	Mean ± SD	95% CI	
True age, y	70.2 ± 9.5	63.4, 71.0	70.2 ± 9.4	69.4, 71.0	0.99
Predicted age, y	68.7 ± 8.8	67.9, 69.4	68.1 ± 8.1	67.4, 68.8	0.27
Prediction difference, y	−1.5 ± 4.5	−1.9, −1.1	−2.1 ± 4.5	−2.5, −1.7	0.040

*p* values are calculated by *t*-test. Prediction difference = Predicted age–True age. SD, standard deviation; CI, confidence interval.

**Table 3:** Age- and gender-adjusted effects of glaucoma on predicted age and prediction difference.

Parameter	Estimate	95% CI	*p* Value	Standard β
Predicted age				
Group, Glaucoma/Control	0.28	0.04, 0.52	0.021 *	0.03
Age, /y	0.78	0.76, 0.81	<0.0001 **	0.88
Gender, Male/Female	−0.17	−0.40, 0.07	0.18	−0.02
Prediction difference				
Group, Glaucoma/Control	0.28	0.04, 0.52	0.021 *	0.06
Age, /y	−0.22	−0.24, −0.19	<0.0001 **	−0.45
Gender, Male/Female	−0.17	−0.40, 0.07	0.18	−0.04

The effects of age and gender are adjusted using multivariate regression analysis based on the least squares fitting method. The R^2^ and adjusted R^2^ are 0.77 and 0.77, respectively, for the predicted age model, and are 0.21 and 0.20, respectively, for the prediction difference model. * and ** indicate *p* < 0.05 and *p* < 0.01, respectively. Prediction difference = Predicted age–True age. CI, confidence interval.

**Table 4:** Comparisons of predicted age and prediction difference between the Glaucoma and control groups across age quartiles.

	Q1 (≤66 y)		Q2 (66–71)		Q3 (71–75)		Q4 (>75 y)	
	Glaucoma	Control	Glaucoma	Control	Glaucoma	Control	Glaucoma	Control
N	140	140	135	135	146	146	126	126
Predicted age, y								
Mean ± SD	58.2 ± 8.8	58.5 ± 8.9	68.7 ± 3.8	68.5 ± 3.5	71.9 ± 3.9	70.8 ± 3.3	76.6 ± 4.4	75.2 ± 3.7
95% CI	56.8, 59.7	57.0, 60.0	68.0, 69.4	67.9, 69.1	71.2, 72.5	70.4, 71.4	75.8, 77.3	74.5, 75.8
*p* value	0.78		0.68		0.015		0.062	
Prediction difference, y								
Mean ± SD	0.3 ± 4.9	0.6 ± 4.8	−0.5 ± 3.6	−0.7 ± 3.6	−1.6 ± 3.8	−2.6 ± 3.2	−4.5 ± 4.0	−5.8 ± 3.8
95% CI	−0.6, 1.1	−0.3, 1.4	−1.1, 0.1	−1.3, −0.1	−2.2, −1.0	−3.2, −2.1	−5.2, −3.8	−6.5, −5.2
*p* value	0.62		0.67		0.013 *		0.0055 **	

*p* values are calculated by *t*-test. * and ** indicate *p* < 0.05 and *p* < 0.01, respectively. Prediction difference = Predicted age–True age. SD, standard deviation; CI, confidence interval.

**Table 5:** Multivariate analysis of systemic parameters associated with age prediction difference.

Parameter	Estimate	95% CI	*p* Value	Standard β	VIF
Group, Glaucoma/Control	0.29	0.05, 0.53	0.019 *	0.06	1.0
Age, /y	−0.22	−0.25, −0.19	<0.0001 **	−0.47	1.4
Gender, Male/Female	−0.19	−0.44, 0.07	0.15	−0.04	1.2
BMI, /kg/m^2^	0.02	−0.06, 0.10	0.59	0.02	1.3
SBP, /mmHg	0.01	−0.00, 0.03	0.21	0.05	1.9
DBP, /mmHg	−0.00	−0.03, 0.03	0.80	−0.01	1.9
HDL-C, /mg/dL	0.01	−0.01, 0.03	0.22	0.04	1.4
LDL-C, /mg/dL	−0.01	−0.02, 0.00	0.08	−0.05	1.1
TG, /mg/dL	−0.00	−0.00, 0.00	0.97	−0.00	1.3
GOT, /U/L	−0.02	−0.06, 0.02	0.38	−0.04	3.3
GPT, /U/L	−0.01	−0.04, 0.03	0.68	−0.02	3.1
γ-GTP, /U/L	0.01	0.00, 0.01	0.036 *	0.07	1.5
Glucose, /mg/dL	−0.00	−0.02, 0.01	0.66	−0.02	2.0
HbA1c, /%	0.29	−0.25, 0.84	0.29	0.04	2.0

The effects of various parameters are adjusted using multivariate regression analysis based on the least squares fitting method. * and ** indicate *p* < 0.05 and *p* < 0.01, respectively. Prediction difference = Predicted age–True age. CI, confidence interval; VIF, variance inflation factor; BMI, body mass index; SBP, systolic blood pressure; DBP, diastolic blood pressure; HDL-C, high-density lipoprotein cholesterol; LDL-C, low-density lipoprotein cholesterol; GOT, glutamic oxaloacetic transaminase; GPT, glutamic pyruvic transaminase; γ-GTP, gamma-glutamyl transpeptidase; HbA1c, glycated hemoglobin.

**Table 6:** Comparisons of fundus vascular changes between the Glaucoma and control groups.

Parameter	Glaucoma	Control	*p* Value
Scheie H			
0	159 (29.1)	239 (43.7)	<0.0001 **
1	356 (65.1)	293 (53.6)	
2	32 (5.9)	14 (2.6)	
3	0 (0)	1 (0.2)	
Scheie S			
0	66 (12.1)	96 (17.6)	0.03 *
1	462 (84.5)	436 (79.7)	
2	19 (3.5)	14 (2.6)	

*p* values are calculated using the extended Fisher’s exact probability test. * and ** indicate *p* < 0.05 and *p* < 0.01, respectively. Scheie H, hypertensive changes; Scheie S, sclerotic changes.

## Data Availability

Data are fully available upon reasonable request to the corresponding author.

## Abbreviations

The following abbreviations are used in this manuscript:

BMI	body mass index
CI	confidence interval
DBP	diastolic blood pressure
γ-GTP	gamma-glutamyl transpeptidase
GOT	glutamic oxaloacetic transaminase
GPT	glutamic pyruvic transaminase
HbA1c	glycated hemoglobin
HDL-C	high-density lipoprotein cholesterol
IOP	intraocular pressure
JPEG	Joint Photographic Experts Group
LDL-C	low-density lipoprotein cholesterol
Q	quartile
SBP	systolic blood pressure
Scheie H	Scheie hypertensive changes
Scheie S	Scheie sclerotic changes
SD	standard deviation
VIF	Variance inflation factor
